# N^6^-Methyladenosine, DNA Repair, and Genome Stability

**DOI:** 10.3389/fmolb.2021.645823

**Published:** 2021-04-09

**Authors:** Fei Qu, Pawlos S. Tsegay, Yuan Liu

**Affiliations:** ^1^ Biochemistry Ph.D. Program; ^2^ Department of Chemistry and Biochemistry; ^3^ Biomolecular Sciences Institute, Florida International University, Miami, FL, United States

**Keywords:** N6-methyladenosine, DNA damage, DNA repair, genome stability, histone modifications

## Abstract

N^6^-methyladenosine (m^6^A) modification in mRNAs and non-coding RNAs is a newly identified epitranscriptomic mark. It provides a fine-tuning of gene expression to serve as a cellular response to endogenous and exogenous stimuli. m^6^A is involved in regulating genes in multiple cellular pathways and functions, including circadian rhythm, cell renewal, differentiation, neurogenesis, immunity, among others. Disruption of m^6^A regulation is associated with cancer, obesity, and immune diseases. Recent studies have shown that m^6^A can be induced by oxidative stress and DNA damage to regulate DNA repair. Also, deficiency of the m^6^A eraser, fat mass obesity-associated protein (FTO) can increase cellular sensitivity to genotoxicants. These findings shed light on the novel roles of m^6^A in modulating DNA repair and genome integrity and stability through responding to DNA damage. In this mini-review, we discuss recent progress in the understanding of a unique role of m^6^As in mRNAs, lncRNAs, and microRNAs in DNA damage response and regulation of DNA repair and genome integrity and instability.

## Introduction

N^6^-methyladenosine (m^6^A) is one of the most abundant epitranscriptomic marks in mRNA of eukaryotic cells that occurs at the consensus motif, RRACH (R is G or A or U, and H is U, A, or C) (Dominissini et al., 2012; Meyer et al., 2012; Zhao et al., 2017). It is regulated by its writer, adenosine methyltransferase, METTL3/METTL14 and erasers, RNA demethylases, fat mass obesity-associated protein (FTO), and Alk B homolog 5 (ALKBH5). m^6^A is usually present at the 5′- and 3′-untranslated regions (5′- and 3′-UTRs) and protein-encoding sequences of mRNAs (Svobodova Kovarikova et al., 2020; Wang et al., 2020). It can also occur in long-noncoding RNAs (lncRNAs) (Meyer et al., 2012), primary microRNAs (pri-miRNA) among others (Iwanami and Brown, 1968; Saneyoshi et al., 1969; Alarcon et al., 2015a; Alarcon et al., 2015b; Yang et al., 2017).

M^6^A in mRNAs was initially discovered by several groups in the 1970s (Desrosiers et al., 1974; Perry and Kelley, 1974). However, its cellular functions have not been extensively explored until 2012, when the methylated RNA immunoprecipitation-sequencing (MeRIP-seq) became available for its genome mapping, and its writers, erasers, and readers were discovered (Meyer and Jaffrey, 2017). In this mini-review, we focus on recent progress in understanding the function of m^6^A in mRNA, lncRNAs, and microRNAs and its unique role in regulating DNA repair and genome stability.

## Mapping of m^6^A in mRNAs

M^6^A can be mapped in a transcriptome-wide manner using MeRIP-seq (Meyer et al., 2012). It can be detected at nucleotide-specific resolution using crosslinking immunoprecipitation (miCLIP)-seq (Linder et al., 2015) during which an anti-m^6^A antibody is UV-crosslinked to the site containing an m^6^A at mRNAs. Subsequently, reverse transcription of mRNAs results in C to T transition or truncation at +1 position relative to m^6^A in cDNAs leading to the mapping of m^6^A located at specific nucleotides. The detection of m^6^A has been further advanced by the newly-developed DART-seq (deamination adjacent to RNA modification targets) from the Meyer group (Meyer, 2019). This approach uses the APOBEC1 fused with the m^6^A-binding YTH domain to induce C-to-U deamination at the sites next to m^6^A. The Us are then detected by RNA-seq. DART-seq is sensitive and specific in mapping m^6^A with a detection limit as low as 10 ng total RNA. It can also quantitatively estimate m^6^A abundance in individual RNA transcripts and determine the dynamic changes of m^6^A induced by genome stressors in cells. The long-read DART-seq is particularly advantageous in mapping the m^6^A profiles in different RNA isoforms. Thus, DART-seq has demonstrated its versatile advantages in mapping transcriptome-wide m^6^A over other approaches.

## m^6^A and Cellular Function and Diseases

### m^6^A Mediates Multiple Cellular Functions

m^6^A can regulate various cellular functions by affecting pre-mRNA splicing (Alarcon et al., 2015a), mRNA degradation (Wang et al., 2014), mRNA and circRNA translation (Wang et al., 2015; Yang et al., 2017), primary microRNA processing (Alarcon et al., 2015a), and protein-RNA interactions (Liu et al., 2015). m^6^A can directly modulate RNA splicing by altering the structures of pre-mRNAs. This increases the accessibility of the heterogeneous nuclear ribonucleoprotein C (hnRNP C) RNA binding motif, facilitating the hnRNP C-RNA interaction and the splicing of pre-mRNA (Liu et al., 2015). m^6^A can also regulate RNA alternative splicing (Alarcon et al., 2015a). Depletion of METTL3 can affect the alternative splicing to alter global gene expression (Alarcon et al., 2015a). Depletion of hnRNPA2B1 with a similar function to METTL3 leads to the same alternation on the global gene expression as METTL3 depletion (Alarcon et al., 2015a). m^6^A can also modulate mRNA stability by reducing the binding of the human antigen R protein (HuR) to the 3′-UTR region of mRNAs and promoting the mRNAs-microRNAs interaction (Wang et al., 2014). m^6^A can regulate mRNA translation through YTHDF1 that binds to m^6^A and help load m^6^A-coded mRNAs to ribosomes, thereby stimulating translation initiation (Wang et al., 2015). On the other hand, m^6^A in circRNA is involved in the initiation of the cap-independent translation. The effect can be further enhanced by METTLE3/14 or diminished by FTO. m^6^A-mediated circRNA translation is also YTHDF3-dependent (Yang et al., 2017). m^6^A in pri-miRNA can promote its conversion to pre-miRNA (Alarcon et al., 2015a). This is accomplished by the recognition of m^6^A by the hnRNPA2B1, promoting its interaction with the DiGeorge Syndrome Critical Region 8 (DGCR8). Subsequently, Drosha, the ribonuclease III enzyme, is recruited to form a microprocessor complex. The hnRNPA2B1-DGCR8 interaction then facilitates DGCR8 binding at the junction between the flanking single-stranded RNA and the stem region in the hairpin structure of pri-miRNA, leading to its cleavage by Drosha (Alarcon et al., 2015a).

### m^6^A is Associated With Diseases

An association between m^6^A and different types of cancer has been implicated. The mutations of m^6^A regulatory genes, METTL3, METTL14, YTHDF1, YTHDF2, FTO, and ALKBH5, have been identified in acute lymphoblastic leukemia, multiple myeloma, and acute myeloid leukemia (AML). Also, copy number variations, predominantly loss of ALKBH5, are found in about 10% of AML patients (Kwok et al., 2017; He et al., 2018). Possible mechanisms by which m^6^A mediates cancer progression are implicated in recent studies. An altered m^6^A level can result in abnormal cellular differentiation in cancer (He et al., 2018). Increased m^6^A in glioma stem-like cells (GSCs) can lead to cellular resistance to radiation through the SOX2-dependent DNA repair (Visvanathan et al., 2018). In addition, m^6^A can mediate the interplay between METTL3 and the oncoprotein HBXIP to stimulate its expression and mediate breast cancer development (Cai et al., 2018). On the other hand, decreased m^6^A promotes endometrial carcinogenesis through an AKT-dependent mechanism by downregulating PHLPP2 and upregulating mTORC2 (Liu et al., 2018). Increased m^6^A reduces AKT activity in the endometrium by enhancing PHLPP2 translation and mTORC2 degradation (Liu et al., 2018). m^6^A can also regulate the stemness of ovarian cancer stem cells by stabilizing the mRNA of the phosphodiesterase, PDE1C, and PDE4B and stimulating the stemness of ovarian cancer cells through the cyclic adenosine monophosphate (cAMP) signaling pathway (Huang et al., 2020). Thus, m^6^A can promote or prevent tumorigenesis upon specific genes and cell types.

## m^6^A Regulates R-Loop formation and Genome Stability

A recent study shows that m^6^A also occurs at the RNA strand of R-loops with a wide distribution at the genome in human pluripotent stem cells (iPSCs) (Abakir et al., 2020). The accumulation of m^6^A on R-loops can exist throughout all the cell cycle phases. Depletion of METTL3 and m^6^A reader, YTH-domain family member 2 (YTHDF2) promotes the accumulation of R-loops, leading to double-strand breaks in iPSCs suggesting that m^6^A prevents the formation of R-loops and DNA damage in iPSCs. Furthermore, m^6^A can lead to the resolution of R-loops induced by DNA damage through the tonicity-responsive enhancer-binding protein (TonEBP). TonEBP binds to R-loops and recruits METTL3 that generates m^6^A on the RNA strand of the R-loop. TonEBP then recruits RNaseH1 to cleave the RNA strand resolving the R-loops (Kang et al., 2021). In contrast, m^6^A in R-loop in some somatic cells promotes the formation of R-loops (Yang et al., 2019). The results suggest that m^6^A plays a distinct role in regulating the formation of R-loops, DNA damage, and genome stability in different types of cells.

m^6^A may also coordinate with ten-eleven translocation 1 (TET1) that can be recruited to R-loops by the growth arrest and DNA damage protein 45A (GADD45A) (Arab et al., 2019) to modulate active DNA demethylation and gene expression. GADD45A can promote active DNA demethylation and TCF21 expression on the R-loop formed at the antisense lncRNA, TARID at the TCF21 promoter (Arab et al., 2019). It is possible that m^6^A on the lncRNA R-loop can facilitate TET1-mediated DNA demethylation leading to the accumulation of DNA demethylation products such as 5hmC. Thus, cells may also coordinate m^6^A with DNA methylation and demethylation to regulate R-loop formation, genomic stability, and gene expression.

## m^6^A Regulates DNA Repair Affecting Genome Stability

### m^6^A Regulates DNA Repair to Modulate Genome Stability

#### m^6^A and UV Damage Repair

m^6^A can be induced by UV irradiation and recruited to UV-damaged sites to promote DNA repair and cellular resistance to UV damage (Xiang et al., 2017). It has been shown that m^6^A generated by METTL3 facilitates UV damage response and repair. This may be mediated by the translesion DNA polymerase κ (Xiang et al., 2017). Moreover, it has been found that upon the UV-induced damage, m^6^A-coded RNAs in the cytoplasm can be translocated back to the nucleus and accumulate at the UV-induced DNA damage sites (Svobodova Kovarikova et al., 2020). It is proposed that m^6^A may guide NER to remove the damage through noncoding RNAs (Svobodova Kovarikova et al., 2020).

#### m^6^A Mediates Double-Strand Break Repair to Prevent Genome Instability

m^6^A can also mediate double-strand DNA (dsDNA) break repair (Zhang et al., 2020). It can be induced by dsDNA breaks through the activation of METTL3, i.e., the phosphorylation at serine 43 (S43) of METTL3 by ATM. m^6^A then recruits YTHDC1 to m^6^A-coded RNAs resulting in the formation of RNA-DNA hybrid at dsDNA breakage sites. Subsequently, RAD51 and BRCA1 are recruited to the dsDNA breaks accomplishing the homologous recombination-mediated dsDNA break repair and preventing genome instability (Zhang et al., 2020).

#### m^6^A and Direct DNA Repair

The role of m^6^A in regulating direct DNA repair is supported by the fact that in mice, m^6^A recognition by YTHDF1 can facilitate the cap-dependent translation (CIT) to stimulate the translation of the direct DNA repair enzyme, O^6^-methylguanine-DNA methyltransferase (MGMT), extending mouse life span (Ozkurede et al., 2019). Moreover, increased METTL3/14 and decreased ALKBH5 and FTO promote the extension of mouse life span (Ozkurede et al., 2019), further suggesting that m^6^A promotes direct DNA repair preventing DNA damage-induced genome instability.

### m^6^A Processing Proteins and DNA Damage and Repair

#### m^6^A and DNA Damage induced by Anti-cancer Drugs

An important research area related to m^6^A is to understand the roles of m^6^A in modulating anti-cancer drug resistance through DNA repair, as this is essentially important for the development of m^6^A regulatory proteins as new anti-cancer drug targets. It has been found that m^6^A increases the stability of the transcription factor-activating enhancer-binding protein 2C (TFAP2C) mRNA, promoting seminoma cell survival under cisplatin treatment. This suggests that DNA repair genes are upregulated by TFAP2C through m^6^A, conferring cellular resistance to the drug-induced DNA damage (Wei et al., 2020). In contrast, decreased m^6^A in β-catenin mRNA promotes the resistance of cervical squamous cell carcinoma to chemo-radiotherapy by upregulating the excision repair cross-complementation group 1 (ERCC1) (Zhou et al., 2018). The results suggest that m^6^A plays a dual role in modulating the resistance of anti-cancer therapies through DNA damage repair upon the types of cancer and their treatments. The cellular pathways that can mediate anti-cancer drug resistance through m^6^A have been discussed in detail in a recent review (Li, B. et al., 2020).

#### m^6^A, FTO, AlkB, and DNA Damage

The roles of m^6^A in regulating the cellular accumulation of DNA damage induced by genotoxicants and their repair can be studied by examining the effects of its writers, readers, and erasers on the cellular sensitivity to DNA damaging agents. It has been shown that FTO knockout in mouse osteoblasts confers the cellular sensitivity to UV and H_2_O_2_, promoting cell death (Zhang et al., 2019). Further analysis demonstrates that FTO erases m^6^A to enhance the stability of mRNAs of Hspa1a and DNA repair genes, thereby protecting cells from DNA damage (Zhang et al., 2019). ALKBH1, on the other hand, has both m^6^A demethylation activity and AP lyase activity that can cleave abasic (AP) sites in DNA through the β-elimination. Since the two types of enzymatic activities share the overlapped active sites (Müller et al., 2017; Müller et al., 2018), m^6^A demethylation in RNA by ALKBH1 may compete with its AP lyase activity resulting in the accumulation of AP sites in the genome. The coordination between the m^6^A demethylation and DNA damage repair of ALKBH1 needs to be further elucidated.

### m^6^A, DNA Damage and Repair, and Bone Development in Mice

m^6^A can also regulate bone development through the modulation of DNA repair. Zhang et al. have shown that mice with FTO global knockout (FTO^*KO*^) or selective knockout in osteoblasts (FTO^Oc^ ^*KO*^) exhibit decreased bone volume in an age-dependent manner. They further demonstrate that FTO KO promotes the accumulation of DNA double-strand breaks in mouse osteoblasts induced by DNA damaging agents and the metabolic stress, high-fat diet while it decreases the level of Hspa1a and Kdm2a protein that is associated with DNA repair (Zhang et al., 2019). These effects promote apoptosis and the death of osteoblasts. The results suggest that the removal of m^6^A by FTO is essential for mammalian osteoblast survival and differentiation, and bone development.

## m^6^A and Its Crosstalk With Histone Modifications

m^6^A can also crosstalk with histone modifications. m^6^A generated in the coding sequence and 3′-UTR can be guided by histone H3 trimethylation at lysine-36 (H3K36me3) and deposited to mRNAs at specific gene loci. METTL14 can bind to H3K36me3 to facilitate the assembly of the m^6^A methyltransferase complex (MTC) that further interacts with RNA polymerase II, thereby stimulating gene locus-specific deposition of m^6^A in nascent mRNA transcripts (Huang et al., 2019). On the other hand, m^6^A also regulates the demethylation of H3K9me2 by recruiting the lysine demethylase 3B (KDM3B) to its target chromatin regions. m^6^A in the chromatin-related mRNAs is bound by YTHDC1, which then recruits KDM3B to its substrate leading to the demethylation of H3K9me2 (Li, Y. et al., 2020). Moreover, m^6^A can interplay with histone modifications by regulating the expression of histone modification enzymes. m^6^A on the mRNA of lysine demethylase 6B (KDM6B) is bound by YTHDF2, causing the degradation of KDM6B mRNAs, reducing the KDM6B protein level, and resulting in a high level of H3K27me3 in proinflammatory cytokine gene like IL6 (Wu et al., 2020). Also, m^6^A can specifically increase the protein level rather than the mRNA level of the histone methyltransferase to stimulate the trimethylation of H3K27 (Chen et al., 2019).

## m^6^A and Telomerase RNA

m^6^A also occurs on the consensus motif, GGACU, located in the duplex region of the secondary structure of human telomerase RNA (hTR) (Han et al., 2020). ALKBH5 can be recruited to hTR to remove m^6^A, and this prevents the assembly of the telomerase complex inhibiting telomerase activity. Thus, m^6^A in hTR can maintain telomere stability by sustaining telomerase activity.

## m^6^A and Regulation of lncRNA and Pri-miRNA

m^6^A in lncRNAs varies upon cell lines, tissue types, and growth stage (Meyer et al., 2012; Han et al., 2019; Xiao et al., 2019). It preferentially occurs in the lncRNAs that undergo alternative splicing (Xiao et al., 2019). m^6^A can mediate the interaction between the large intergenic coding RNA 1281 (linc1281) and the pluripotency-related let-7 family miRNAs to sequester the miRNAs leading to maintenance of mouse embryonic stem cell identity (Yang et al., 2018). m^6^A at specific sites of the X-inactive specific transcript (XIST) mediates the transcriptional silencing of the genes on the X chromosome by recruiting YTHDC1, promoting XIST-regulated gene repression (Patil et al., 2016).

Also, m^6^A can modulate the interaction between lncRNAs and their binding proteins. m^6^A in the metastasis-associated lung adenocarcinoma transcript 1 (MALAT1) promotes the binding of hnRNPG, hnRNPC, and METTL16 to the MALAT1 transcripts, thereby altering the level of the lncRNA (Zhou et al., 2016). m^6^A in the Olfr29-ps1 present in myeloid-derived suppressor cells (MDSCs) plays a significant role in MDSC immunosuppression and differentiation by stimulating the production and stability of Olfr29-ps1 (Shang et al., 2019).

m^6^A on the promoter-associated non-coding RNA-D (pncRNA-D) plays a critical role in regulating cell cycle progression by modulating the interaction between the RNA and the fused in sarcoma/translocated (FUS/TLS) protein in liposarcoma (Yoneda et al., 2020). m^6^A recruits YTHDC1 to prevent the FUS/TLS protein from binding to pncRNA-D, thereby increasing the cyclin D1 expression.

m^6^A can be deposited to the pri-miRNA of microRNA, which is processed by the microprocessor complex of the pri-miRNAs that contains the RNA binding protein DGCR8 and Drosha type III ribonuclease (Denli et al., 2004; Gregory et al., 2004; Han et al., 2004; Landthaler et al., 2004). m^6^A on pri-miRNA is bound by hnRNPA2B1 protein that recruits DGCR8 stimulating nuclear miRNA processing (Alarcon et al., 2015a). METTL3 depletion reduces the binding of DGCR8 to pri-miRNAs resulting in the decrease of matured miRNAs and accumulation of unprocessed pri-miRNAs (Alarcon et al., 2015b). METTL14 can directly recruit DCGR8 on the m^6^A-coded pri-miRNA to stimulate the expression of the oncosuppressor, miR-126a, in hepatocellular carcinoma (HCC) (Ma et al., 2017). This suggests that m^6^A governs HCC metastasis by regulating miRNA levels. m^6^A can also modulate microRNA expression by regulating the stability of the AGO2 mRNA and increasing the AGO2 protein level (Min et al., 2018). In addition, m^6^A is involved in regulating the oncogenic miR-25-3p in pancreatic duct epithelial cells induced by cigarette smoke condensate (CSC) (Zhang et al., 2019). CSC can cause the hypomethylation at the promoter region of METTL3 to upregulate the methyltransferase, increasing m^6^A. Subsequently, this upregulates miR-25-3p, thereby promoting malignant phenotypes of pancreatic cancer cells (Zhang et al., 2019).

## Discussion

**FIGURE 1:**
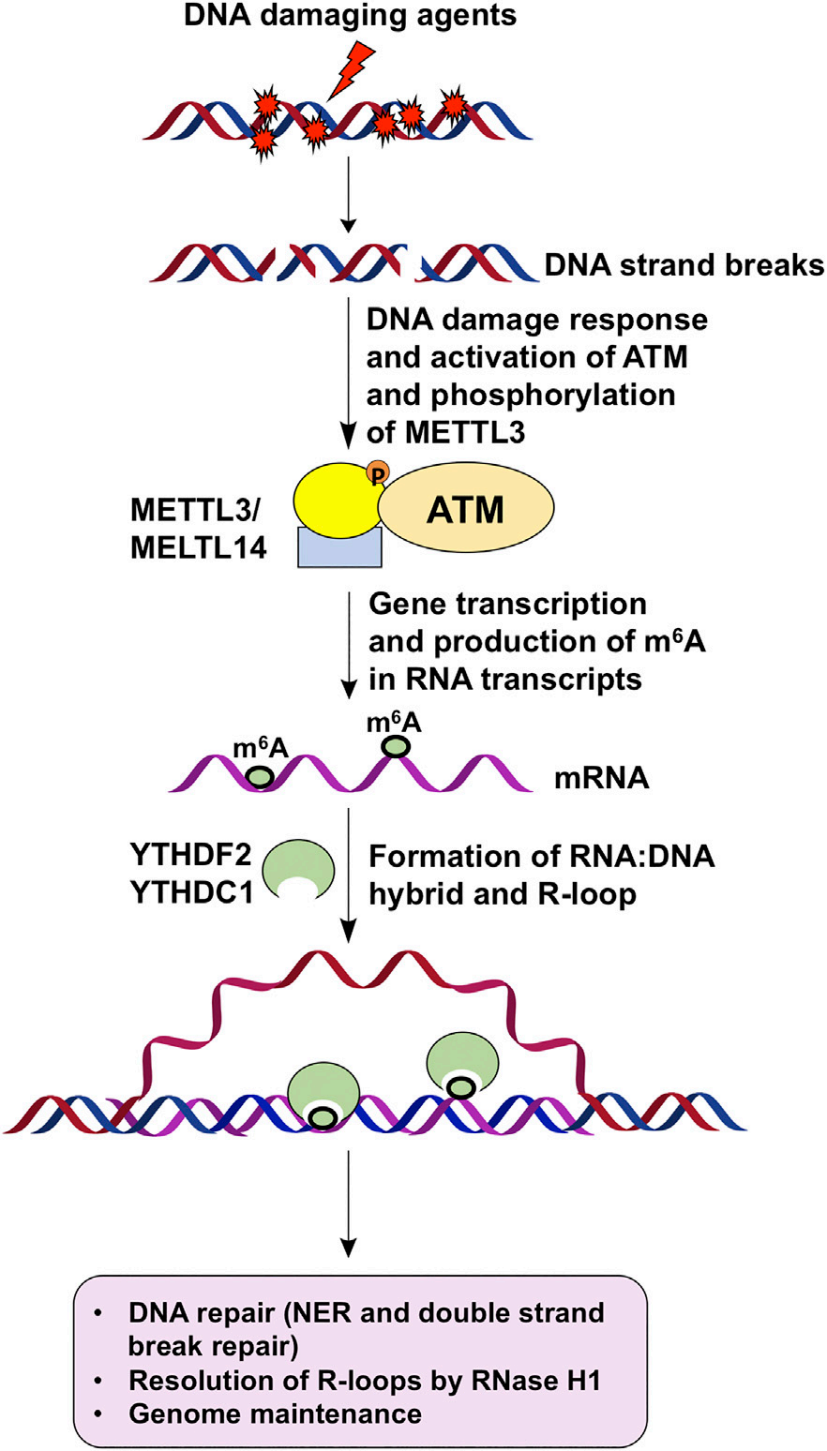
m^6^A regulates DNA repair to maintain genome stability via R-loops. DNA damage agents induce strand breaks to activate DNA damage response and ATM, which phosphorylates and activates METTL3. DNA damage response then leads to gene transcription and production of m^6^A by activated METTL3 on mRNA transcripts. Subsequently, m^6^A-coated mRNAs hybrid with their DNA template to form R-loops. m^6^A readers such as YTHDF2 and YTHDC1 then bind to m^6^A on R-loops to facilitate DNA repair and resolution of R-loops through RNase H1, thereby leading to genome maintenance.

Current studies support that m^6^A is involved in DNA repair and genome maintenance in an R-loop-dependent manner (Figure 1). However, Our understanding of the multidimensional roles of m^6^A in regulating DNA repair and genome stability is just in its infancy. An important question that needs to be addressed is the differential roles of m^6^A and its regulators in mediating diversified DNA damage response and repair pathways that maintain genome stability. Another outstanding question that needs to be elucidated in the future is the interplay among m^6^A, noncoding RNAs, and DNA and histone epigenetics in modulating DNA repair and genome stability. In addition, a fast and straightforward high throughput platform and technology need to be developed for determining the stoichiometry of m^6^A and other RNA modifications that coexist in RNA transcripts. These future studies should significantly advance our understanding of the molecular mechanisms underlying m^6^A-mediated DNA repair, genome stability, and cellular function.
